# Estimation of intracardiac shunts in young children with a novel indicator dilution technology

**DOI:** 10.1038/s41598-020-58347-2

**Published:** 2020-01-28

**Authors:** Theodor Skuli Sigurdsson, Lars Lindberg

**Affiliations:** 1 Department of Pediatric Anesthesia and Intensive Care, Children´s Hospital, University Hospital of Lund, Lund, Sweden; 2 Department of Anesthesia and Intensive Care, Landspítalinn, University Hospital of Iceland, Reykjavík, Iceland

**Keywords:** Circulation, Paediatric research

## Abstract

Clinical evaluation of intracardiac shunts in children is not straightforward. Echocardiography can only diagnose the presence of a shunt but does not estimate the shunt ratio. This can be a critical factor that influences treatment options. In this single-center, prospective, observational, method-comparison study, we validate the ability of a novel monitoring device COstatus to estimate the intracardiac shunt ratio (Qp/Qs) of pulmonary (Qp) to systemic (Qs) blood flow in young children before and after corrective cardiac surgery. The indicator dilution technology COstatus monitor was compared to two other more invasive reference techniques, perivascular ultrasonic flow probes (placed around the pulmonary truncus and ascending aorta) and the oximetric shunt equation (using arterial and venous blood gases). Our study revealed that the COstatus monitor detected intracardiac shunts with high sensitivity and specificity but there was some underestimation of the shunt ratios compared to the reference techniques.

## Introduction

Clinical evaluation of critically ill children by physicians is often imprecise and can lead to an unreliable estimation of the true hemodynamic condition^1–5^. A reliable hemodynamic monitoring device that is safe and easily adapted in children would be beneficial to guide physicians in clinical decisions^6–8^. The diagnosis and evaluation of an intracardiac shunt in a critically ill child is also important in regards to treatment, as undiagnosed congenital heart disease with intracardiac shunt has been associated with significant morbidity^9^. In left-to-right shunts, cardiac defects at the atrial level (atrial septal defects and anomalous pulmonary venous drainage) cause oxygen-saturated blood to reenter the pulmonary circulation resulting in increased right heart volume load. On the other hand, cardiac defects at the ventricular or great artery level (ventricular septal defects, patent arterial duct and aortopulmonary collaterals) may cause either left heart volume overload or a combination of left heart volume and right heart pressure overload (depending on the size of the defect). In right-to-left shunts, oxygen-desaturated blood bypasses the pulmonary circulation and causes arterial desaturation. This can be seen in children with large intracardiac defects, where saturated and desaturated blood is mixed, in children with atrial septal defects with high central venous pressure or in children with ventricular septal defects with high right ventricular pressure, such as those with pulmonary hypertension. The difference in pulmonary (Qp) and aortic blood flow (Qs) is one of the variables used to estimate the size of intracardiac shunts (by calculating the Qp/Qs ratio) as a determination of clinical significance and indication for surgical correction (Table 1).

**Table 1:** Definition of shunts depending on the quotient or ratio between the blood flow in pulmonary circulation (Q_P_) and systemic circulation (Q_S_): Qp/Qs.

Q_P_/Q_S_	Shunt type
<1.0	Right-to-left shunt
1.0	No shunt
1.0 < x < 1.5	Small left-to-right shunt
1.5 ≤ x < 2.0	Moderate left-to-right shunt
≥2.0	Large left-to-right shunt

The methods used to determine the Qp/Qs ratio are usually highly invasive and require general anesthesia. In the catheterization laboratory, it is possible to retrieve blood gases from arterial and mixed venous blood and use the oximetric shunt formula to calculate a Qp/Qs ratio. There are non-invasive methods to diagnose intracardiac shunts available, but they are imprecise and have clinical limitations^10^. Echocardiography has become the method of choice to detect the presence of intracardiac shunts but is not accurate in determining the Qp/Qs ratio. Magnetic resonance imaging can estimate the Qp/Qs ratio but is impractical in critically ill children and is time consuming.

COstatus (Transonic Systems Inc, Ithaca, NY, USA) is a novel monitoring device that can detect shunts and estimate the Qp/Qs ratio by analyzing transcardiopulmonary blood dilution with saline detected by ultrasound sensors^11^. The technique is also able to determine cardiac output, total end-diastolic cardiac volume, central blood volume and active circulation blood volume, all of which can be of value in the evaluation of a critically ill child^12–17^.

The primary aim of this study was to determine how accurate COstatus was in discriminating between the presence of a shunt and no shunt. The secondary aim was to compare the Qp/Qs ratio of the intracardiac shunts calculated by COstatus with two other methodologically different reference techniques. With the use of perivascular ultrasonic transit time flow probes placed around the pulmonary truncus and aorta, the pulmonary blood flow (Qp) and aortic blood flow (Qs) could be simultaneously measured to determine the Qp/Qs ratio. The Qp/Qs ratio was also calculated by the oximetric shunt formula using collected arterial and venous blood gases.

## Results

A total of forty-four children were included in the study. The mean age of the patients was 12 months (range 1 to 43 months), and the mean weight was 7.1 kg (range 2.7 to 13.6 kg). The surgeon was not able to place the perivascular flow probe in one child without compromising circulatory stability, resulting in 215 paired COstatus and perivascular flow probe estimations (PVFP) of Qp/Qs ratios (five for each child) and 44 consecutive oximetric shunt equation (OSE) calculations of Qp/Qs ratios before and after the surgical correction (estimated mean Qp/Qs ratios are shown in Table 2).

**Table 2:** Estimated mean Qp/Qs ratios by COstatus (n = 5), perivascular flow probes (n = 5) and oximetric calculations (n = 1) before the surgical correction.

Patient	Defect	Qp/Qs			Patient	Defect	Qp/Qs		
		COstatus	PVFP	OSE			COstatus	PVFP	OSE
**1**	ASD/VSD	BD	2.0	1.4	**23**	ASD	>2	4.9	4.2
**2**	VSD	>2	2.4	2.9	**24**	ASD	1–1.5	4.3	2.9
**3**	VSD	>2	3.2	3.9	**25**	VSD	>2	3.5	3.7
**4**	ASD	>2	M	7.3	**26**	ASD/VSD	>2	9.7	10.0
**5**	ASD	>2	3.4	3.9	**27**	ASD/VSD	>2	3.9	2.9
**6**	VSD	1.5–2	2.8	2.4	**28**	ASD	>2	3.8	2.3
**7**	VSD	1–1.5	3.6	3.0	**29**	VSD	>2	2.0	3.3
**8**	VSD	1.5–2	3.6	2.9	**30**	VSD	1.5–2	2.0	4.5
**9**	VSD	1–1.5	3.2	2.5	**31**	VSD	>2	3.0	2.5
**10**	VSD	1.5–2	3.6	3.8	**32**	ASD/VSD	>2	1.9	3.5
**11**	VSD	1.5–2	2.6	3.9	**33**	ASD/VSD	>2	2.4	2.4
**12**	VSD	1.5–2	2.3	2.6	**34**	VSD	1–1.5	1.6	1.5
**13**	VSD	1.5–2	3.0	4.4	**35**	ASD	1–1.5	2.1	1.1
**14**	ASD	>2	2.6	2.0	**36**	VSD	1–1.5	1.8	1.4
**15**	VSD	>2	3.9	3.7	**37**	VSD	1–1.5	1.3	1.4
**16**	ASD	>2	1.6	2.3	**38**	VSD	>2	4.6	3.2
**17**	VSD	1.5–2	2.8	4.5	**39**	ASD/VSD	>2	2.1	1.4
**18**	ASD	1	1.7	1.3	**40**	ASD/VSD	>2	3.4	2.8
**19**	ASD	>2	3.1	2.4	**41**	ASD/VSD	>2	1.8	2.5
**20**	VSD	1–1.5	1.4	1.7	**42**	ASD	BD	3.1	2.7
**21**	VSD	1.5–2	2.4	3.0	**43**	VSD	1–1.5	1.0	2.0
**22**	ASD/VSD	1.5–2	4.3	4.3	**44**	VSD	1–1.5	1.3	3.1

PVFP = perivascular flow probe, OSE = oximetric shunt equation.

ASD = atrial septal defect, VSD = ventricular septal defect.

BD = bidirectional shunt, M = missing value.

COstatus detected a Qp/Qs ratio of >1.0 in 43 out of 44 patients with a transesophageal echocardiography (TEE) confirmed defect before the surgical correction. In 2 out of 44 patients, COstatus detected an intracardiac bidirectional shunt. These two patients were excluded from the comparison of left-to-right shunts between the techniques.

The mean Qp/Qs _PVFP_ was 2.9 ± 1.2 (range 1.0 to 9.7), and the mean Qp/Qs _OSE_ was 3.0 ± 1.6 (range 1.1 to 10.0). The bias between Qp/Qs _PVFP_ vs Qp/Qs _OSE_ was 0.07 using Bland-Altman analysis (LOA ± 1.84), while the percentage error between Qp/Qs _PVFP_ vs Qp/Qs _OSE_ was 58.8%.

**Figure 1:**
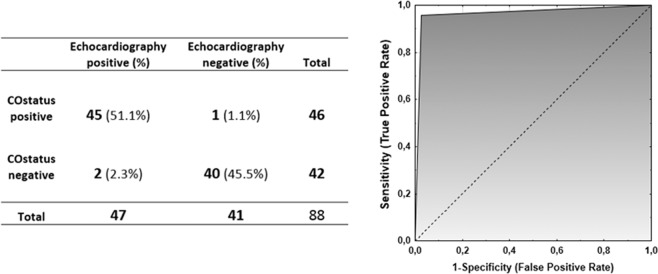
Comparison between COstatus and transesophageal echocardiography for detecting and excluding a left-to-right shunt and an ROC curve showing accuracy for diagnosing a left-to-right shunt.

Three very small residual defects were detected by color flow doppler signals of the TEE after surgical correction. Two of them were also indicated by Qp/Qs ratios >1.0 by COstatus. In addition, COstatus also indicated a small residual shunt in another patient (supported by the oximetric Qp/Qs calculation) but not detected by the color flow doppler of the TEE. This resulted in a sensitivity of 95.7% (95% CI: 85.5% to 99.5%) and specificity of 97.6% (95% CI: 87.1% to 99.9%) for COstatus to discriminate between intracardiac left-to-right shunt and no shunt. The area under the receiver operating characteristic (ROC) curve was 0.97 to diagnose an intracardiac left-to-right shunt (Fig. 1).

**Figure 2:**
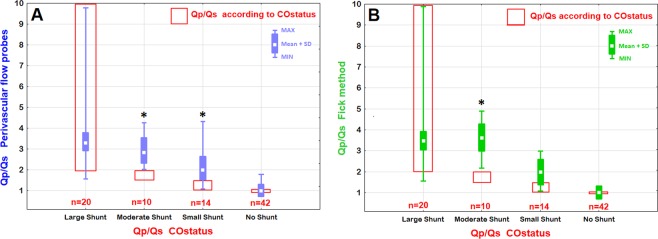
Comparison between categorized groups of Qp/Qs ratios of COstatus and Qp/Qs ratios estimated by perivascular flow probes around the pulmonary truncus and aorta (**A**) and Qp/Qs ratios calculated by the oximetric shunt equation from blood gas analysis. (**B**) The vertical bars contain 95% confidence intervals in each categorized shunt group (^*^indicates P < 0.05).

The categorized Qp/Qs ratios estimated by COstatus showed a statistically significant underestimation (p < 0.05) for the Qp/Qs ratios in moderate and small shunt size groups compared with Qp/Qs determined by the perivascular flow probes and those calculated by the oximetric shunt equation (Fig. 2). Since COstatus only gives a value of Qp/Qs ratio >2 in the large shunt size group (shown in Table 2) it is not possible to comment on the exact accuracy compared with the other techniques in the large shunt size group.

## Discussion

In the present study, we found that the COstatus monitoring device detects intracardiac left-to-right shunts in most cases, but there was some underestimation regarding shunt ratios compared to the reference methods.

Our results agree with earlier studies that have compared COstatus with oximetric calculation of the Qp/Qs ratio during cardiac catheterization and in an animal model, in which COstatus detects intracardiac left-to-right shunt with high sensitivity and specificity^18,19^. The specificity in this study was probably higher than in the article by Saxena *et al*., due to a high number of tiny intracardiac left-to-right shunts of no clinical significance undetected by echocardiography in their study^15^. The incidence of residual shunt after surgical correction was low and only detected in 3 patients (7%) by postoperative TEE. These very small intracardiac left-to-right shunts were all considered clinically insignificant. In one case, the COstatus was unable to detect a left-to-right blood flow (only detected in one out of five measurements), although it was observed by the color flow doppler. Sometimes the pressure gradient between the atrium may be low and varying in time, making the blood flow across the septum minimal and therefore difficult to detect by dilution technology. On the other hand, COstatus detected a very small left-to-right blood flow together with the oximetric calculation, which was not observed by the TEE, indicating a high degree of sensitivity for detection of intracardiac left-to-right shunts, even those that were clinically insignificant or difficult to detect by echocardiography.

There was a statistically significant underestimation of the shunt ratio as shown in Fig. 2 in moderate and small shunt size groups in this study compared to the reference methods. Although Fig. 2 seems to show a good agreement between all methods in the large shunt size group, Table 2 reveals that COstatus only indicates a large shunt with a Qp/Qs ratio >2 which makes it difficult to interpret shunt accuracy between methods in the large shunt size group. This underestimation might partly be explained by Transonic’s approach in formulation of the algorithm to calculate the shunt ratio. The perivascular flow probe around the ascending aorta does not include the coronary blood flow and may therefore give a slightly to low Qs and thus an inaccurate high Qp/Qs ratio. The shape of the dilution curve is dependent on heart rate, ventricular function and peripheral resistance, which may cause difficulties in the formulation of a general algorithm for all ages, ventricular functions, shunt sizes and level of blood volumes. Our results might hint that a possible improvement of the algorithm may be needed for shunt ratio determination in this young age group. This underestimation is in contrast with earlier studies, where shunt estimation with dilution methods have overestimated the Qp/Qs ratios in children compared with oximetric techniques^20–23^. The overestimation of Qp in these studies may be caused by the rapid systemic recirculation that occurs in children. This resulted from the technique that was used at the time to determine the area under curve (AUC) providing an inaccurate overestimation of the AUC, which reflects Qp. In adults, the oximetric technique has provided Qp/Qs shunt estimates that were larger than the dilution technique, which is in agreement with our findings^24^. The estimation of Qp/Qs by the oximetric technique agreed well with the estimation made by the flow probes with only a difference of 0.07 in mean Qp/Qs ratio but with a relative high percentage error (58.8%). Direct blood gas measurements included in the oximetric shunt equation involved calculating venous oxygen saturation by adding three times the oxygenation saturation in the superior vena cava with saturation in the inferior vena cava and dividing the sum by four (Flamm’s formula). This could result in a false low estimation of venous oxygen saturation, explaining possible differences between methods. An alternative explanation for the disagreement between methods is that equilibrium between the oxygen uptake and the oxygen consumption did not exist. The quantification of Qp/Qs ratios may be of value to determine the need for surgical correction. However, since COstatus presents intracardiac left-to-right shunts as a range value in accordance with clinical significance, it was difficult to perform a true validation of how well the Qp/Qs ratios were estimated or how large the shunt actually was in compared with the other methods.

One limitation of the study is that it was technically impossible to have two flow probes placed around both the ascending aorta and the pulmonary truncus in the infants at the same time as it affected the blood flow in the large vessels and coronaries. Another important limitation is that the aortic flow probe reading does not account for the myocardial blood flow and thus always underestimates systemic blood flow (Qs). The amount of coronary blood flow is uncertain but has been claimed to be approximately 7% (probably higher in a heart with a left-to-right shunt due to myocardial strain). The oximetric technique always relies on the assumption of similar oxygen uptake and consumption and that the relation between the oxygen saturation in the inferior and superior caval vein is constant. This study only looked at shunt estimations in simple intracardiac shunts (ASD and/or VSD defects). It remains to be seen how well COstatus performs in patients with more complex mixing lesions (e.g. single ventricles). The final limitation is that the arterial line was not available for continuous arterial pressure monitoring during COstatus measurement sessions.

The strength of this study is the high number of homogenous younger patients compared to that of previous hemodynamic pediatric studies and that measurements were performed simultaneously with all methods within a very narrow time period.

The technology of the COstatus monitor is less invasive compared to previous cardiac output reference methods (e.g. cardiac catheterization, thermodilution, perivascular flow probes and the Fick method) and is highly sensitive and specific regarding detection and estimation of left-to-right shunts (an extra feature not available in earlier bedside hemodynamic monitors). It is easily applied in young children with less technical limitations, as it only requires a central and an arterial line (often in place in critically ill patients), uses body-temperature saline as an indicator in small amounts, involves no blood loss and does not require invasive control of ventilation or sedation/anesthesia when catheters are in place. COstatus has performed well in pediatric cardiac output comparison studies and could help bridge the gap between invasive hemodynamic monitors and non-invasive methods in the future^12,25–27^. In our opinion, the COstatus monitor is not a technique that can replace echocardiography in the evaluation and the clinical decision-making regarding need for cardiac intervention. However, used in combination with echocardiography, it adds to important information regarding general hemodynamic status in patients, including cardiac output.

To summarize, the COstatus monitoring device detected intracardiac shunts with high sensitivity and specificity, but was not superior to echocardiography. There was some underestimation of shunt ratios with COstatus compared with the reference techniques.

## Methods

### Patient and data collection

Patients enrolled in this study were part of a cohort of patients undergoing elective cardiac surgery for the correction of an atrial septal defect (ASD) and/or a ventricular septal defect (VSD). The inclusion criteria included informed written parental consent, weight <15 kg, and ASD and/or VSD defect. Children with conditions that influenced the stability of the measurements, such as perioperative arrythmias and/or valvular regurgitations, were excluded. The study was approved by the Ethics Committee of Lund University, Sweden (Dnr 2013636).

### Experimental protocol

Anesthesia was induced using fentanyl (5 mcg/kg) and penthothal (5 mg/kg) and maintained with isoflurane (0.5–1.0%). Pancuronium (0.2 mg/kg) was given to facilitate intubation with a cuffed endotracheal tube. All children received a peripheral arterial catheter in the radial artery (Neoflow 24 G in <5 kg patients and Venflow 22 G in >5 kg patients; BD Ltd, Wokingham, UK) and a central venous catheter (Multicath triple lumen 6 cm, 4.5 F; Vygon Ltd, Swindon, UK) in the right internal jugular vein. The COstatus AV loop was connected to the arterial catheter and to the distal lumen of the central venous catheter. The AV loop was primed with heparinized (2 units/ml) normothermic isotonic saline. COstatus ultrasound sensors were placed on the arterial and venous sides of the AV loop. A preoperative transesophageal echocardiography (TEE) was performed to determine cardiac function and existing defects. Prior to cardiopulmonary bypass, the surgeon placed perivascular flow probes consecutively around the ascending aorta and then the pulmonary truncus. The size of the probes made it impossible to fit them both simultaneously without compromising the circulation. Five consecutive measurements were performed of ascending aortic blood flow simultaneously with five measurements with COstatus. This was immediately followed by five consecutive measurements of pulmonary blood flow. Thereafter, the surgeon took blood samples from the pulmonary truncus, inferior vena cava (IVC), and superior vena cava (SVC) by direct puncture. An arterial blood sample was simultaneously taken from the arterial catheter, and the Qp/Qs ratio was calculated by the oximetric shunt formula. The fraction of inspiratory oxygen was kept at 0.3–0.4 to ensure full arterial oxygen saturation and, therefore, fully saturated blood in the left atrium. After the surgical correction, the measurements were repeated in the same order. The surgical result was controlled by a postoperative TEE investigation by a cardiologist who was blinded to the other findings.

The algorithm in COstatus estimates a Qp/Qs ratio in every patient. Transonic has chosen to present it as a range value in accordance with clinical significance. The children were thus categorized into five different groups of Qp/Qs ratios (Table 1).

### Shunt measurements

#### COstatus^®^

**Figure 3:**
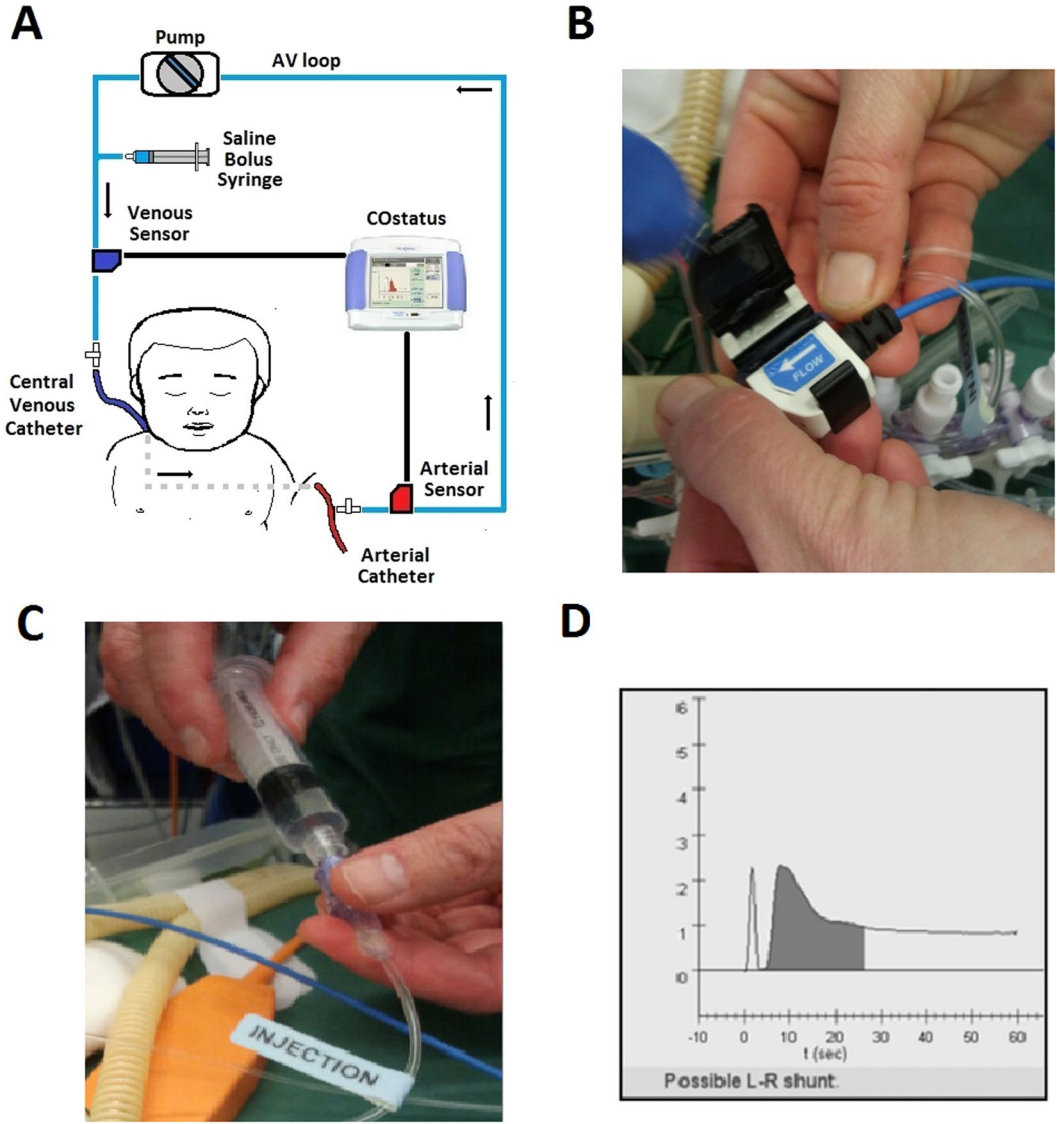
(**A**) Schematic picture of COstatus monitor and AV loop setup connected to a patient, (**B**) placement of venous ultrasound probe on AV loop, (**C**) injection of saline bolus into the AV loop, and (**D**) arterial dilution curve screen display showing a possible shunt.

**Figure 4:**
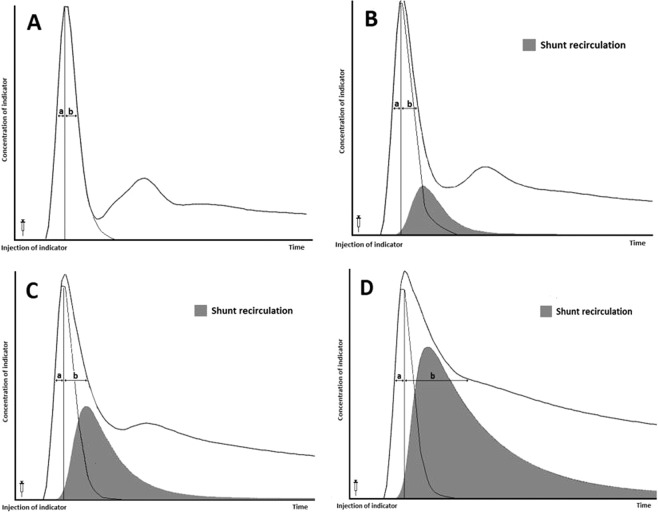
The effect of a left-to-right intracardiac shunt observed in the arterial curve. One part of the injected indicator (saline) passes normally through the cardiopulmonary system and exits into the aorta. The second part recirculates multiple times through the pulmonary system (gray area) until all indicators exit the heart. This delay is observed in the descending part of the arterial dilution curve. (**A**) Normal dilution curve without intracardiac shunt, (**B**) Small intracardiac left-to right shunt, (**C**) Moderate intracardiac left-to-right shunt and (**D**) Large left-to-right intracardiac shunt. By analyzing the distance between an imaginary vertical line from peak dilution and the ascending dilution curve (a) and the descending dilution curve (b) at the half peak level, an asymmetry can be detected indicating the presence of a possible left-to-right shunt. (Pictures are adapted from the Shunts Modeling program, Transonic Systems Inc.).

The COstatus monitor device (Transonic Systems Inc., Ithaca, NY, USA) uses a specific single-use extracorporeal arteriovenous circuit (AV-loop), which is connected to the arterial and central venous catheters already in place (Fig. 3A). External ultrasound sensors are then attached to the AV loop to detect the level of blood dilution (Fig. 3B). A roller-pump connected to the AV-loop guarantees a constant blood flow rate of 9–12 ml/min. A determined volume (0.5–1.0 ml/kg) of isotonic saline, warmed to body temperature, is injected into a venous port near the venous ultrasound sensor, which is connected to the AV-loop (Fig. 3C). As blood and saline have different ultrasound velocities (blood has an ultrasound velocity of 1.560–1.585 m/s and saline 1.530 m/s), there will be a reduction in ultrasound velocity when saline is added to the circulation. This change in ultrasound velocity (sometimes called ultrasound dilution) can then be used to estimate cardiac output and other hemodynamic parameters (just as temperature changes are used to estimate cardiac output in thermodilution). Venous ultrasound sensors measure the ultrasound velocity of blood when it passes through the venous part of the AV loop just before entering the circulation via the central venous catheter. Then, in turn, the arterial ultrasound sensor connected to the arterial part of the AV-loop detects changes in the ultrasound velocity of the blood as it exits the arterial line after transcardiopulmonary passage. This transient decrease in ultrasound velocity produces both a venous and arterial dilution curve that are then compared and analyzed by the COstatus monitor (Fig. 3D). The systemic dilution of the injected volume of saline is then used to calculate the ejected cardiac output by modifying the Stewart-Hamilton equation^28–30^. In a normal heart without intracardiac shunts, an undistorted and almost symmetric dilution curve is detected by the arterial sensor. Then, the diluted blood returns to the arterial sensor after a second systemic recirculation. This is recognized by a second lower bump occurring later in the arterial dilution curve trace in a biventricular circulation without shunt defects. This systemic recirculation is rapid in children and may complicate shunt detection. In the presence of a left-to-right intracardiac shunt, the diluted blood reenters the pulmonary circulation through the defect. This results in a delay of a full flush of the saline from the cardiopulmonary system and is observed in the descent of the declining limb of the arterial dilution curve (Fig. 4). The delay in the declining part of the curve depends on the amount of shunted blood that recirculates. In defects with a large shunt, the amount of blood that reenters the pulmonary circulation may be so substantial that it merges with the diluted blood that reappears after the second systemic recirculation. This will affect the downward slope of the declining limb and the total curve area, which makes an adequate calculation of the Qp/Qs ratio difficult.

Using an imaginary vertical line from the peak dilution (peak change in ultrasound velocity) to the base, the symmetry or asymmetry of the descending part can be detected at the half peak level, by comparing the distance between the vertical line and the ascending curve line (a) and between the vertical line and the descending curve line (b) (Fig. 4). According to research data, the a:b ratio in a four-chambered heart without a shunt is approximately 1.4 ± 0.13 (or ±13%). This variation is associated with factors such as the rate of injection, accuracy of the algorithm to calculate area, and other hemodynamic factors that can cause asymmetry in the dilution curve besides shunts. Since the ascending part of the curve reflects the dilution without pulmonary recirculation, it can be used to construct an almost similar imaginary descending curve with a delay less than 1:1.4. The area under that imaginary curve (AUC) and the Stewart-Hamilton principle can then be used to calculate a plausible normal blood flow without recirculation. Then, the AUC and the Stewart-Hamilton principle can be used to calculate the blood flow beneath the actual curve containing the recirculated blood flow corresponding to the delayed descending curve. The difference between these curves corresponds to the shunt recirculation leading to a calculation of the Qp/Qs ≈ (b/a)/1.4 ± 13% displayed on the screen (Fig. 4B). In our data analysis, COstatus was considered to have identified a shunt if we observed the screen message (Fig. 3D) in at least 2 out of the 5 consecutive measurements.

### Perivascular flow probes (PVFP)

**Figure 5:**
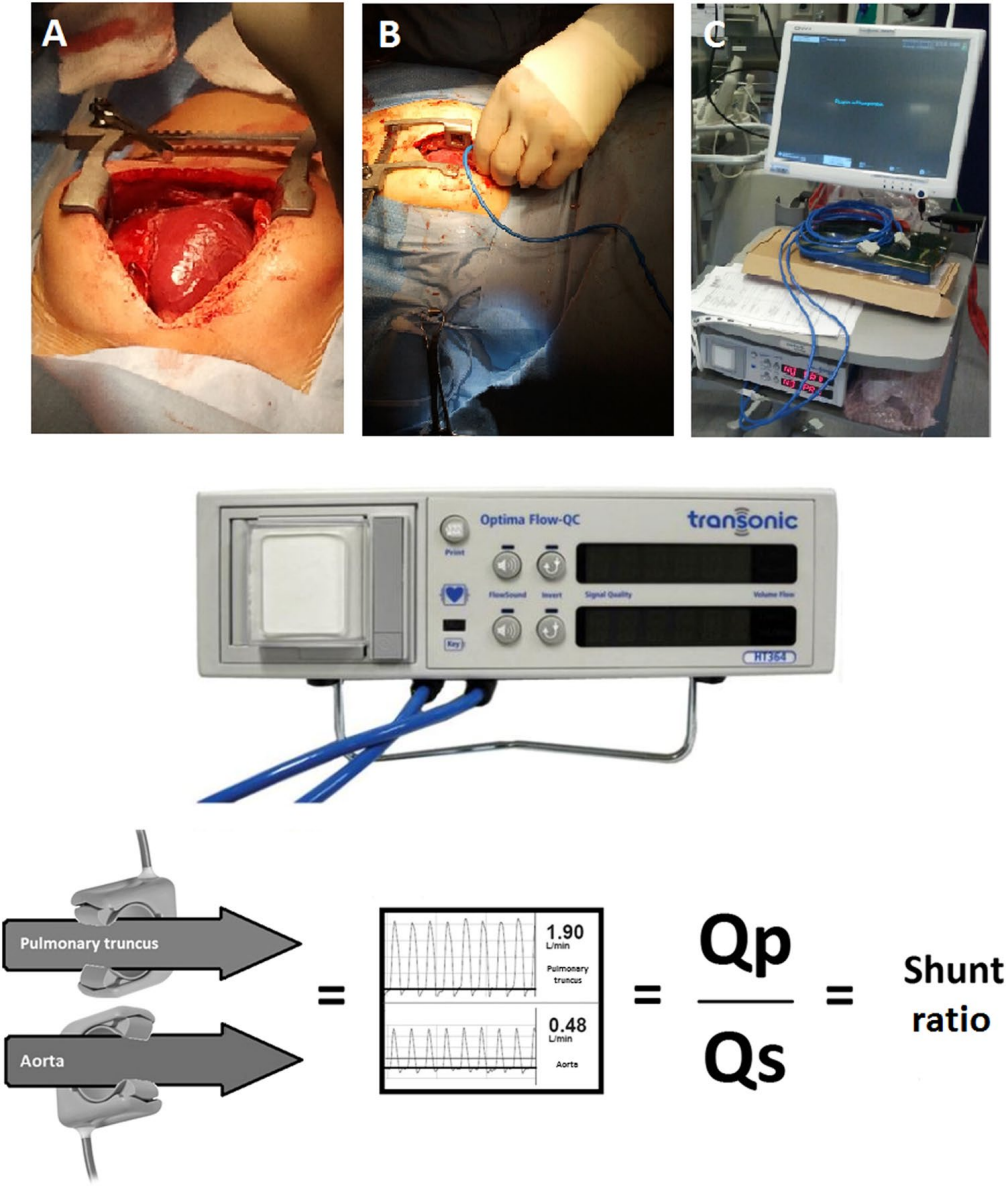
Optima dual channel Flow-Qc meter, model HT364 and a schematic explanation of how the invasive COnfidence ultrasound flow probes were used to compare differences in blood flow in both pulmonary truncus (Qp) and ascending aorta (Qs) to estimate the shunt ratio (Qp/Qs). (**A**) Exposure of the heart through an open sternotomy, (**B**) placement of flow probe on the great arteries for blood flow measurements, and (**C**) in-theatre setup for the diagnostic AureFlow monitor and Optima flow meter. (Photos of flow probes and flow meter adapted with permission of Transonic Systems Inc.).

Perivascular ultrasonic transit-time flow probes, COnfidence, AU-series (Transonic Systems Inc., Ithaca, NY, USA) were positioned around the pulmonary truncus and around the ascending aorta distal to the branch of the coronary arteries. These flow probes were custom designed (available in different diameter sizes) to fit around vessels to measure real-time blood flow. The flow probes come pre-calibrated from the manufacturer, with a certified length of use for more than one year and can be used multiple times as they tolerate standard sterilization. When the flow probes had been placed correctly, they were connected to Optima dual channel Flow-Qc meter, model HT364 (Transonic Systems Inc, Ithaca, NY, USA) and the diagnostic software AureFlo (Transonic Systems Inc, Ithaca, NY, USA), which provides a graphic display of the blood flow. By comparing blood flow in the pulmonary truncus (Qp) and ascending aorta (Qs), the shunt ratio can be calculated by dividing Qp with Qs (Qp/Qs) (Fig. 5). Transit-time technology has been used and validated in earlier studies and is now considered an established reference method for cardiac output^16,31,32^.

### Oximetric shunt equation (OSE)

In patients with left-to-right shunts, the oximetric shunt equation can be used to estimate the ratio between pulmonary blood flow (Qp) and systemic blood flow (Qs), Qp/Qs. By assuming that the oxygen uptake (VO2) in Eq. 1 is similar to the oxygen consumption (VO2) in Eq. 2, the shunt fraction Qp/Qs can be derived from Eq. 3. (*CaO*_2_ = content of oxygen in arterial blood, *CmvO*_2_ = content of oxygen in venous blood (by Flamm’s formula (3SVC + IVC)/4), *CpvO*_2_ = content of oxygen in pulmonary venous blood, and *CpaO*_2_ = content of oxygen in pulmonary arterial blood.

1 $$Qp\,(L/\,{\rm{\min }})=\frac{V{O}_{2}\,(ml/min)}{Cpv\,{O}_{2}\,(ml/L)-Cpa\,{O}_{2}\,(ml/L)}$$

2 $$Qs\,(L/\,{\rm{\min }})=\frac{V{O}_{2}\,(ml/min)}{Ca\,{O}_{2}\,(ml/L)-Cmv\,{O}_{2}\,(ml/L)}$$

3 $$Shunt\,ratio=\frac{Qp}{Qs}=\frac{Ca\,{O}_{2}-Cmv\,{O}_{2}}{Cpv\,{O}_{2}-Cpa\,{O}_{2}}$$

In this study, blood gas analysis was performed with the ABL800 Flex Radiometer (Radiometer AS, Brønshøj, Denmark). The pulmonary venous oxygen saturation was expected to be full since the arterial oxygen saturation was 97–100%.

### Transesophageal echocardiography

A Philips S8–3T pediatric transesophageal echocardiography transducer probe was used in this study with a Philips iE33 ultrasound system (Philips Healthcare, Andover, MA, USA) for pre- and postoperative evaluation of cardiac function. Conventional cross-sectional echocardiography and doppler color flow were used to detect the defects. The size of the defect was estimated and categorized as follows:

**Very small:** if there was only a narrow jet of flow detected by the color flow Doppler.

**Small:** if there was a wider jet on the color flow doppler and the defect could be visualized on 2D imaging.

**Moderate to Large:** if the flow was clearly visualized by the color flow doppler, the defect could be visualized on 2D imaging, and there were visual signs of right cardiac chamber overload.

### Statistical analysis

The statistical power analysis was performed for sample size estimation based on data from an earlier pilot study comparing Qp/Qs ratios (14). In that study, the bias was 0.4, and the pooled SD was 0.7, resulting in an effect size of 0.57. G-Power 3.1.9.3 software (Kiel University, Germany) was used for power calculations. To reject the null hypothesis, the sample size for this study with an alpha error of p 0.05 and power of 0.90 needed to be at least n = 28. The final number of patients was related to the fact that a higher number of patients was needed for a simultaneous cardiac output comparison study^25^.

Histograms and Shapiro-Wilk test were used to confirm normal distribution of the data.

Analysis of variance (ANOVA) was used to detect statistical significance between the three different techniques used to determine Qp/Qs ratios. The bias between Qp/Qs ratios by the perivascular flow probes (Qp/Qs _PVFP_) and the oximetric saturation data (Qp/Qs _OSE_) was analyzed according to the Bland-Altman method, accounting for multiple measurements per individual^33,34^. The mean difference (bias) was calculated and plotted against the average of the comparison. The 95% limits of agreement (LOA) were calculated as the mean bias of 1.96* ± SD (standard deviation of the difference between methods). The percentage error (PE) was calculated using the following equation:

$$PE=\frac{1.96\ast SDbias}{mean\,PVFP\,(\frac{Qp}{Qs})}\ast (100 \% )$$

Statistica version 13 (Dell Inc., Tulsa, OK, USA) was used for statistical analysis, and all data are expressed as the mean ± standard deviation (SD) unless otherwise indicated.

### Ethical standards

This study was approved by the Ethics Committee of Lund University, Sweden, and was in accordance with the ethical standards laid down in the 1964 Declaration of Helsinki and its later amendments.

### Informed consent

Written informed consent was obtained from the parents of all patients.
